# A prognostic model for thermal ablation of benign thyroid nodules based on interpretable machine learning

**DOI:** 10.3389/fendo.2024.1433192

**Published:** 2024-08-19

**Authors:** Zuolin Li, Wei Nie, Qingfa Liu, Min Lin, Xiaolian Li, Jiantang Zhang, Tengfu Liu, Yongluo Deng, Shuiping Li

**Affiliations:** ^1^ Department of Ultrasound, Longyan First Affiliated Hospital of Fujian Medical University, Longyan, China; ^2^ Department of Information, Longyan First Affiliated Hospital of Fujian Medical University, Longyan, China; ^3^ School of Information Engineering, Minxi Vocational & Technical College, Longyan, China

**Keywords:** artificial intelligence, benign thyroid nodule, machine learning, thyroid nodules, thermal ablation, ultrasound-guided, volume reduction rate

## Abstract

**Introduction:**

The detection rate of benign thyroid nodules is increasing every year, with some affected patients experiencing symptoms. Ultrasound-guided thermal ablation can reduce the volume of nodules to alleviate symptoms. As the degree and speed of lesion absorption vary greatly between individuals, an effective model to predict curative effect after ablation is lacking. This study aims to predict the efficacy of ultrasound-guided thermal ablation for benign thyroid nodules using machine learning and explain the characteristics affecting the nodule volume reduction ratio (VRR).

**Design:**

Prospective study

**Patients:**

The clinical and ultrasonic characteristics of patients who underwent ultrasound-guided thermal ablation of benign thyroid nodules at our hospital between January 2020 and January 2023 were recorded.

**Measurements:**

Six machine learning models (logistic regression, support vector machine, decision tree, random forest, eXtreme Gradient Boosting [XGBoost], and Light Gradient Boosting Machine [LGBM]) were constructed to predict efficacy; the effectiveness of each model was evaluated, and the optimal model selected. SHapley Additive exPlanations (SHAP) was used to visualize the decision process of the optimal model and analyze the characteristics affecting the VRR.

**Results:**

In total, 518 benign thyroid nodules were included: 356 in the satisfactory group (VRR ≥70% 1 year after operation) and 162 in the unsatisfactory group. The optimal XGBoost model predicted satisfactory efficacy with 78.9% accuracy, 88.8% precision, 79.8% recall rate, an F1 value of 0.84 F1, and an area under the curve of 0.86. The top five characteristics that affected VRRs were the proportion of solid components < 20%, initial nodule volume, blood flow score, peripheral blood flow pattern, and proportion of solid components 50–80%.

**Conclusions:**

The models, based on interpretable machine learning, predicted the VRR after thermal ablation for benign thyroid nodules, which provided a reference for preoperative treatment decisions.

## 1 Introduction

With advancements in imaging technology, the detection rate of thyroid nodules has increased annually, with the detection of benign thyroid nodules exceeding 90% (1). Benign thyroid nodules can cause local eminences that affect appearance and require intervention when patients experience compression or anxiety. Ultrasound-guided thermal ablation has been shown to shrink nodules and preserve thyroid function (2) and is recommended in the relevant treatment guidelines (3–5). Volume reduction ratio (VRR) is a direct index for evaluating the clinical efficacy of ablation (6–8). The degree and speed of lesion absorption after ablation varies greatly among individuals. Additionally, effective predictive models that analyze the factors influencing the efficacy of benign thyroid nodule ablation are limited.

Since the advent of the big data era, machine learning has gradually become widely used in the medical field owing to its huge advantages in analyzing and processing massive datasets, including disease diagnoses, drug production, and medical data analyses (9). A previous study showed that machine learning models based on the ultrasonic characteristics of benign thyroid nodules can predict radiofrequency ablation (RFA) efficacy (10) and assist clinicians in formulating appropriate treatment plans.

Nevertheless, to our knowledge, no studies have been conducted on the prediction of efficacy for patients with benign thyroid nodules using models based on clinical and ultrasonic characteristics. The traditional machine learning model often has “black box” characteristics; therefore, it is difficult to explain the internal mechanism of the model and the basis of the prediction results. Therefore, this study aimed to build several machine learning models based on multi-directional data, identify a suitable prediction model, analyze the characteristics affecting therapeutic outcomes through interpretable technology, and provide a reference for clinical decision-making.

## 2 Materials and methods

### 2.1 Patient data and ethical approval

The clinical and ultrasonic image data of patients who underwent RFA or microwave ablation (MWA) for benign thyroid nodules under ultrasound guidance at the Longyan First Hospital between January 2020 and January 2023 were analyzed. The inclusion criteria were as follows: 1) thyroid ultrasonography showing thyroid nodules and pathological results of two fine needle aspiration biopsies showing benign nodules; 2) maximum diameter of thyroid nodule ≥2 cm, accompanied by compression symptoms or cosmetic problems; 3) voluntary ablation treatment; and 4) 12-month outpatient follow-ups.

The exclusion criteria were the following: 1) previous history of radioactive iodine therapy or thermal ablation therapy; 2) incomplete or missing clinical and follow-up data; 3) ablation lesion with two or more fused nodules, and 4) poor image quality, making evaluations impossible.

All patients provided informed consent for the use of their medical record data. The study was approved by the Medical Ethics Committee of the Longyan First Affiliated Hospital of Fujian Medical University (approval number: [2020] Ethics Committee Approval for Scientific Research No. 042) and the Chinese Clinical Registration Center (registration number: ChiCTR2100041923). The research process was undertaken in line with the Helsinki Declaration.

### 2.2 Ultrasound-guided thermal ablation of thyroid nodules

An experienced interventional ultrasound physician performed the ablation procedures using ultrasound guidance during the operation. Routine disinfection, covering, and local infiltration anesthesia were performed. The isolation zone was formed by injecting a 20–40 mL mixture of 0.5% lidocaine hydrochloride and normal saline between the thyroid membrane and the surrounding structures. The set power range was 30–35 W.

For solid thyroid nodules, the “moving shooting” technique for layer-by-layer ablation of nodules and “leverage pry-off” method to assist ablation for nodules in the dangerous triangle area were used. After ablation, contrast-enhanced ultrasound was conducted to perform supplementary ablation of the remaining lesions in the perfusion area.

**Figure 1:**
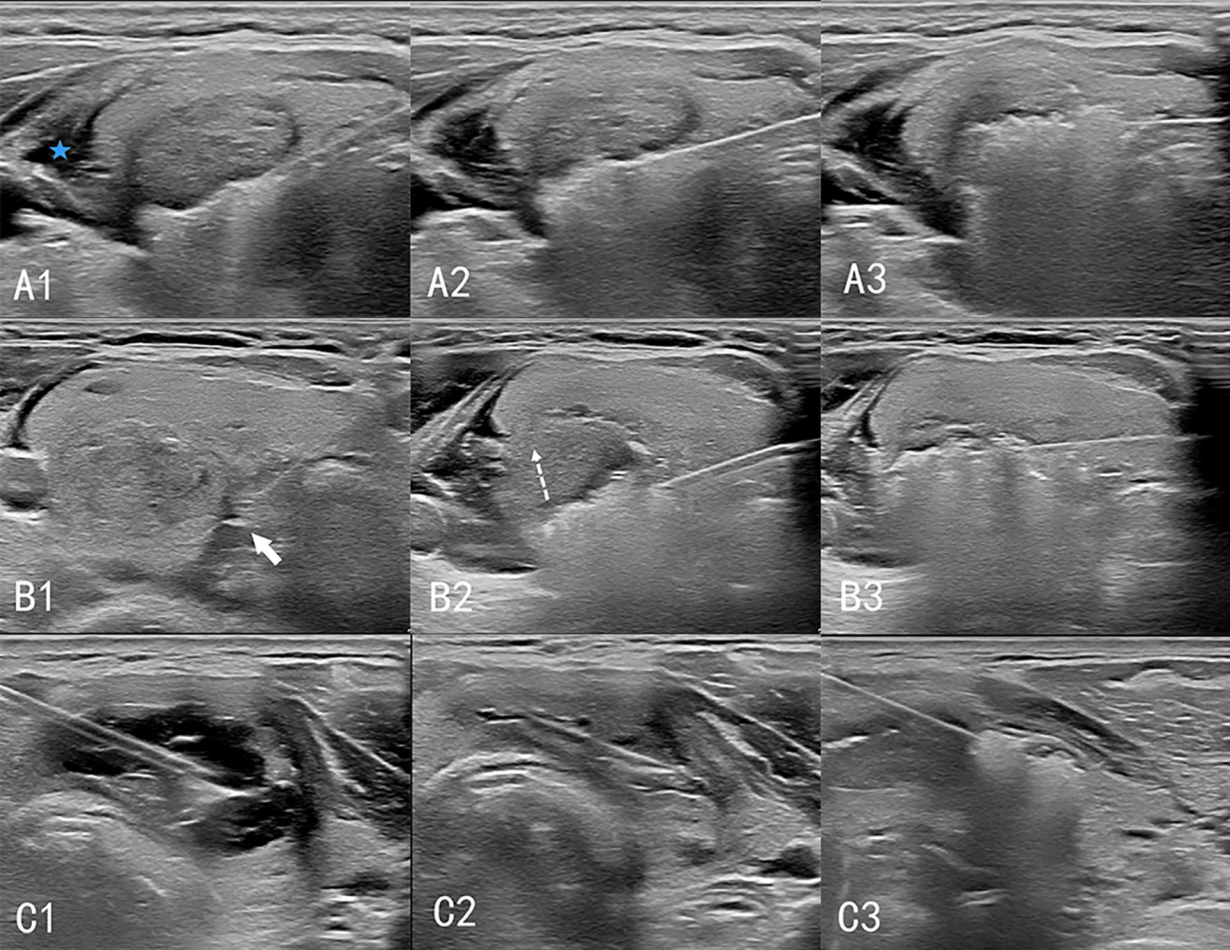
Procedure of ultrasound-guided thermal ablation of thyroid gland. **A1–A3**: Ablation of solid nodules. The isolation zone (blue 5-pointed star) is established, and the “moving shooting technique” is used for ablation from deep to shallow. **B1–B3**: Ablation of high-risk nodules. **(B1)** A spacer (white arrow) is injected between the thyroid and the trachea to separate the thyroid from the tissue surrounding the danger triangle. **(B2)** When ablating lesions near the danger triangle, the “leverage pry-off method” is used to lift the needle and pull the tissue upward (dashed arrow) to prevent heat damage to the surrounding tissue. **(B3)** Ablation of the lesion layer-by-layer. **C1–C3**: Cystic nodular ablation. **(C1)** A 50 mL syringe needle is inserted into the cystic area of the nodules for fluid extraction. **(C2)** The nodules are considered solid nodules after drainage. **(C3)** Ablation is performed according to the procedure for solid nodules.

Cystic nodules were treated as solid nodules after the cyst fluid was extracted from them; thermal ablation and postoperative management were performed according to the steps for solid nodules (**Figure 1**).

### 2.3 Data collection

Patient demographic information, including name, sex, and age; previous and present history, including whether accompanied by Hashimoto’s thyroiditis (HT); test data including routine blood, coagulation function, and thyroid function; and ultrasonic characteristics were collected.

The features were independently extracted from ultrasound images by two senior sonographers. For each patient, the extracted information of the largest nodule was used for the analysis. Any differences were resolved through negotiation; if required, another senior physician was invited to discuss any unresolved differences to reach a consensus. Eight signs were analyzed: 1) the positions of the nodules, divided into left lobe, isthmus, and right lobe; 2) the degree of risk of the nodule location, according to the anatomical position and relationships to the surrounding organs, was divided into high risk and not high risk. High risk was defined as a distance of less than 2 mm between the nodule edge and the trachea or esophagus (11).; 3) proportion of solid components: < 20%, 20%–50%, 50%–80%, and ≥ 80%; 4) echo, including hypoechoic, medium echoic, and hyperechoic; 5) initial nodule volume (cm³) = 0.52 × upper and lower diameters (cm) × left and right diameters (cm) × anteroposterior diameter (cm); 6) blood flow distribution pattern was divided into peripheral dominant, internal dominant, and both peripheral and internal; 7) blood flow score on a five-point scale: 0 points (no color signal observed); 1 point (small amount of speckled color signal); 2 points (color signal<25%); 3 points (color signals account for 25–50%); 4 points (color signal>50%); and 8) calcification and strong echo spots larger than 2 mm, accompanied by sound shadows. Personal information irrelevant to the predictions was excluded from the study. A total of 55 clinical and ultrasonographic features were analyzed.

### 2.4 Efficacy evaluation criteria

In this study, the average VRR after 12 months of ablation was 73.75 ± 23.3%, therefore we used 70% as the cut off for a satisfactory result. One year after the operation a VRR ≥70% was defined as satisfactory and <70% was defined as unsatisfactory. VRR was calculated using the following formula: VRR = (initial nodular volume − ablation volume)/initial nodular volume ×100%.

### 2.5 Data and preprocessing

There were 518 benign thyroid nodules: 356 with a satisfactory curative effect and 162 with an unsatisfactory curative effect. First, features were cleaned and classified variables (such as sex) were converted into virtual variables. Missing data were filled with the K-nearest neighbor. Then the data was normalized and standardized. The standardized data were screened out by single factor correlation to screen out the top 10 features that influenced the prediction results. The sample was divided into training and test datasets at a ratio of 7:3, and each feature was standardized. The Synthetic Minority Oversampling Technique was used to accommodate for the imbalance of training data.

### 2.6 Model construction

The tools used in the model-building process were Python 3.8 and scikit-learn1.2.2. (Python Software Foundation, Beaverton, OR, USA). Logistic regression, support vector machine, decision tree, random forest (RF), eXtreme Gradient Boosting (XGBoost), and Light Gradient Boosting Machine (LGBM) algorithms were used to construct a predictive model for satisfactory efficacy after ablation. The tuning of the model evaluated all possible combinations of hyperparameters using the grid search method and determined the best hyperparameter to establish the area under the curve (AUC) of the receiver operating characteristic (ROC) for each model. Test data were used to evaluate the model performance, including accuracy, precision, recall rate, F1 score, and AUC. The AUC was used as the main evaluation indicator to select the best-performing prediction model. The SHapley Additive exPlanations (SHAP) method was used to quantify the contribution of each feature for the optimal model output to achieve model interpretability and analyze the predictors that affected the VRR. The flow chart of this method is illustrated in **Supplementary Figure S1**.

### 2.7 Statistical analyses

Statistical analyses were performed using IBM SPSS Statistics 26 software (IBM Corp., Armonk, NY, USA). All measurement data are described as mean ± standard deviation (x̄ ± SD). Comparisons between groups were performed using the independent sample t-test or the Mann–Whitney U test. Count data are expressed as an example (%), and the chi-square test or Fisher’s exact probability method was used for comparisons between groups. Statistical significance was set at *P* < 0. 05.

## 3 Results

### 3.1 Baseline patient characteristics

The baseline patient characteristics are presented in **Table 1**. A total of 518 nodules were included. The satisfactory and unsatisfactory treatment groups comprised 356 and 162 patients, respectively. The mean age of the patients was 47.42 ± 12.00 years. The average initial nodule volume was 12.00 ± 12.64 mL. The average VRR at 12 months was 73.75 ± 23.3%. There were no significant differences in age, body mass index, ablation type, nodule location, calcification, combination with Hashimoto’s thyroiditis, or thyroid stimulating hormone (thyrotropin), free triiodothyronine, or free thyroxine levels between the groups (*P>*0.05). There were significant differences between the two groups regarding sex, initial nodule volume, nodule echo, proportion of solid components, risk degree for nodule location, blood flow distribution pattern, and blood flow score (*P<*0.05).

**Table 1:** Baseline data of patients and nodules.

Characteristic	Satisfactory group (n=162)	Unsatisfactory group (n=356)	*P*-value
Age (years)	46.66 ± 12.33	47.77 ± 11.86	0.334
Sex/n (%)			0.006
Male	14(8.6%)	64(18.0%)	
Female	148(91.4%)	292(82.0%)	
BMI (kg/m^2^)	22.98 ± 2.89	23.18 ± 3.10	0.530
Ablation type/n (%)			0.384
MWA	111(68.5%)	230(64.6%)	
RFA	51(31.5%)	126(35.4%)	
Initial nodule volume (ml)	14.86 ± 12.20	10.70 ± 12.65	0.001
Nodule location/n (%)			0.855
Left lobe	77(47.5%)	170(47.8%)	
Right lobe	80(49.4%)	178(50.0%)	
Isthmus	5(3.1%)	8(2.2%)	
Proportion of solid components			<0.001
<20%	1(0.6%)	58(16.3%)	<0.05
20%–50%	11(6.8%)	65(18.3%)	<0.05
50%–80%	46(28.4%)	80(22.5%)	<0.05
≥80%	104(64.2%)	153(43.0%)	>0.05
Echo/n (%)			0.045
Hypoechoic	55(34.0%)	137(38.5%)	<0.05
Medium echoic	88(54.3%)	199(55.9%)	>0.05
Hyperechoic	19(11.7%)	20(5.6%)	>0.05
Risk degree of nodule location/n (%)			0.021
High risk	60(37.0%)	96(27.0%)	
Not high risk	102(63.0%)	260(73.0%)	
Blood flow distribution pattern			<0.001
Peripheral dominant	15(9.3%)	99(27.8%)	<0.05
Internal dominant	9(5.6%)	9(2.5%)	>0.05
Both peripheral and internal	138(85.2%)	248(69.7%)	<0.05
Blood flow score	2.24 ± 0.81	1.79 ± 0.83	<0.001
Calcification/n (%)			0.105
No	13(8.0%)	16(4.5%)	
Yes	149(92.0%)	340(95.5%)	
Combined with HT/n (%)			0.384
No	145(89.5%)	327(91.9%)	
Yes	17(10.5%)	29(8.1%)	
TSH/(mIU/L)	1.23 ± 1.10	1.37 ± 1.15	0.170
FT3/(pmol/L)	5.58 ± 1.24	5.65 ± 1.23	0.936
FT4/(pmol/L)	15.60 ± 5.14	15.73 ± 5.13	0.450

BMI, body mass index; MWA, microwave ablation; RFA, radiofrequency ablation; HT, Hashimoto’s thyroiditis; TSH, thyroid stimulating hormone (thyrotropin); FT3, free triiodothyronine; FT4, free thyroxine. This table only lists some of the features used in the test; 55 features were used in the actual test.

### 3.2 Efficiency evaluation of the model

**Figure 2:**
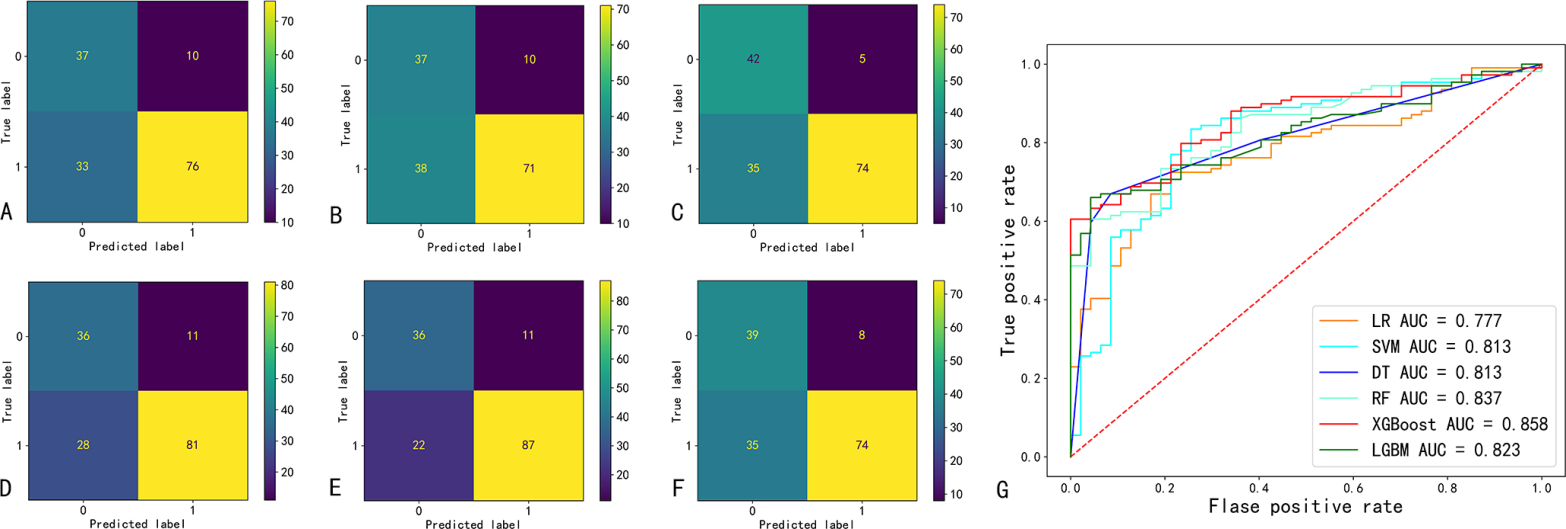
Confusion matrix and ROC curve of six machine learning prediction models. **(A-F)** indicate the confusion matrix of the logistic regression (LR), support vector machine (SVM), decision tree (DT), random forest (RF), extreme Gradient Boosting (XGBoost), and eXtreme Gradient Boosting (LGBM); respectively. **(G)** ROC curves and AUC values of six machine learning models. AUC, area under the curve of the receiver operating characteristic (ROC).

The confusion matrix and ROC curve of the six machine learning models in the test set are shown in **Figure 2**, and the prediction efficiencies are listed in **Table 2**. The XGBoost model exhibited the highest prediction accuracy (78.9%), F1 score (0.84), and AUC value (0.86).

**Table 2:** Predictive effectiveness for the six types of machine learning.

Index	LR	SVM	DT	RF	XG Boost	LGBM
Accuracy	72.4%	69.2%	74.4%	75.0%	78.9%	72.4%
Precision	88.4%	87.7%	93.7%	88.0%	88.8%	90.2%
Recall rate	69.7%	65.1%	67.9%	74.3%	79.8%	67.9%
F1 score	0.78	0.75	0.79	0.81	0.84	0.77
AUC	0.77	0.81	0.81	0.84	0.86	0.82

LR, logistic regression; SVM, support vector machine; DT, decision tree; RF, random forest; XGBoost: extreme Gradient Boosting; LGBM, Light Gradient Boosting Machine; AUC, area under curve of the receiver operating.

### 3.3 Feature importance analysis

**Figure 3:**
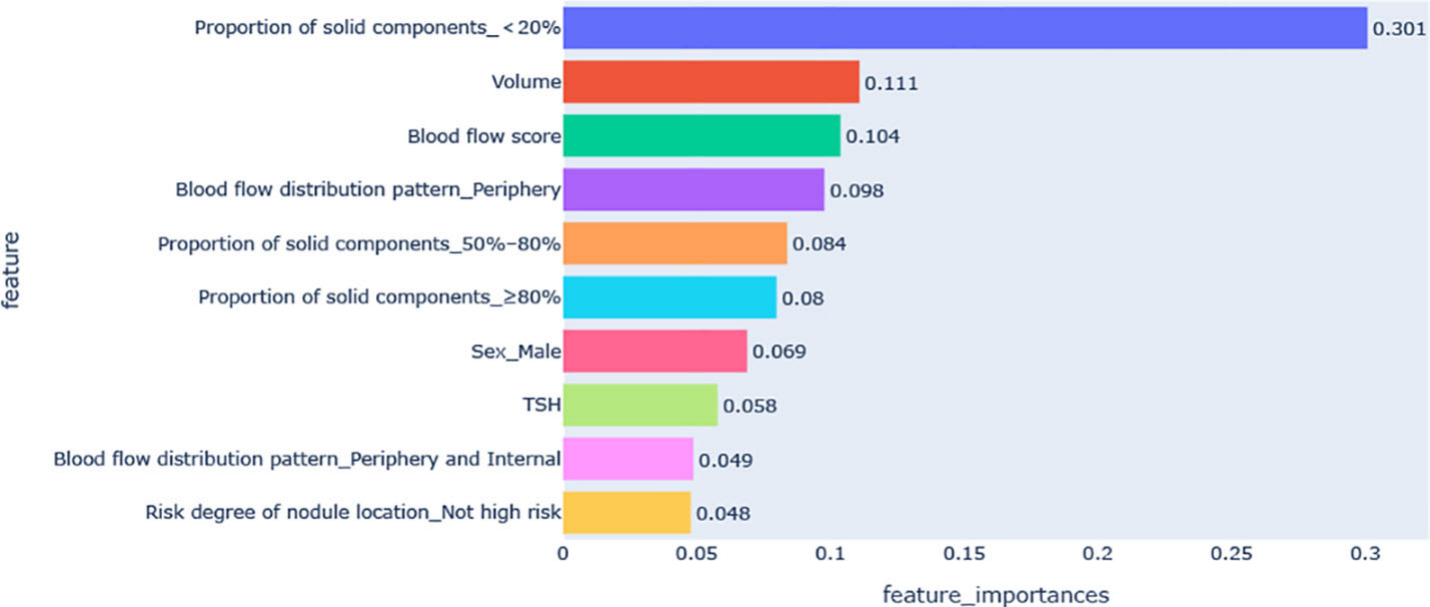
Feature importance diagram of the optimal model. TSH, thyroid stimulating hormone (thyrotropin).

**Figure 3** shows the feature importance analysis of the SHAP algorithm for the optimal XGBoost model. The importance of the Shapley feature is measured by the average Shapley absolute value – the larger the value, the greater the contribution to the model prediction results. Among them, the proportion of solid components < 20% was the most important feature affecting VRR ≥70%, followed by volume, blood flow score, peripheral blood flow, and the proportion of solid components 50%–80%; the influence of features on the model output decreases in turn.

**Figure 4:**
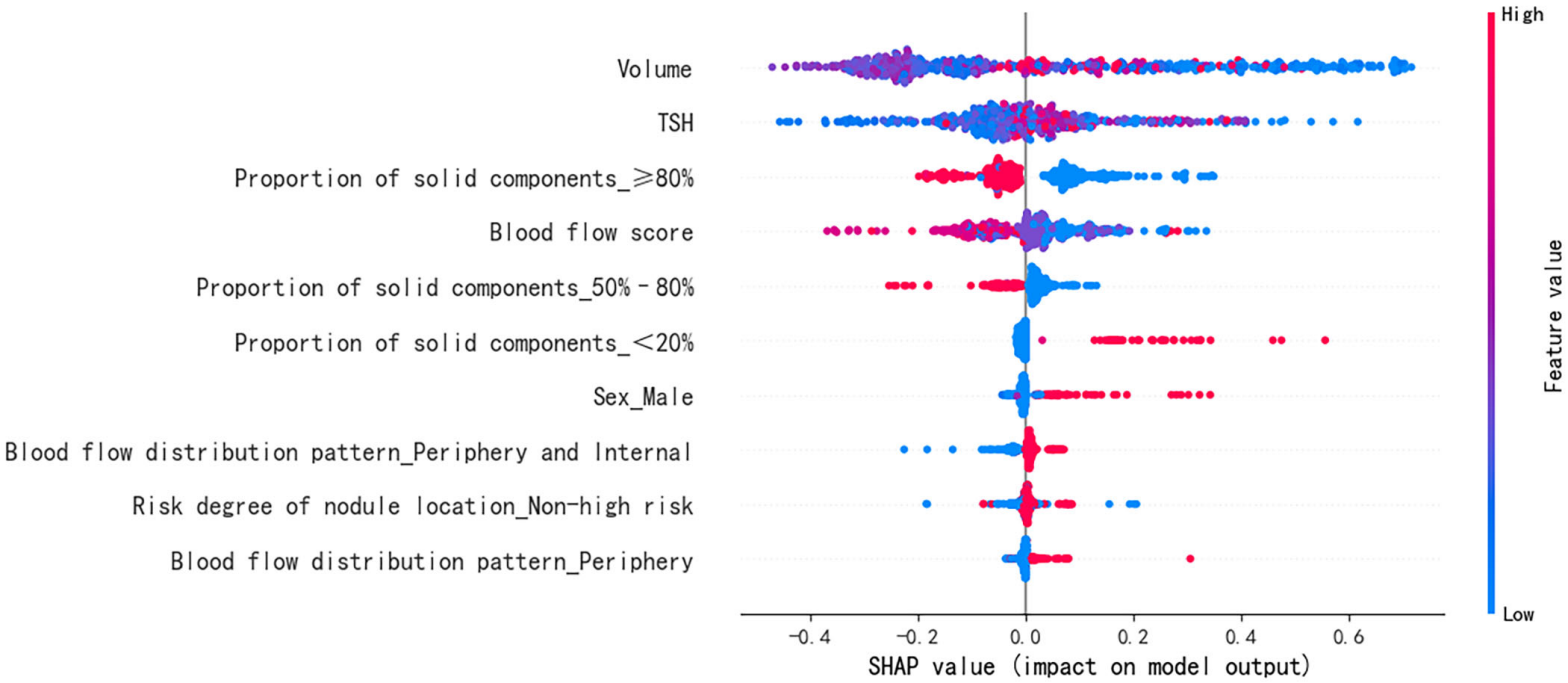
Feature SHAP diagram of the optimal model. The horizontal axis marks the SHAP value. A positive value indicates positive prediction whereas a negative value indicates negative prediction. The vertical axis shows the features included in the model, ranked in descending order of importance according to their impact on the predicted results. Each point in the figure represents a sample, and the color of the point represents the original value of the feature. The closer the point is to red, the larger the value; the closer the point is to blue, the smaller the value. SHAP, SHapley Additive exPlanations; TSH, thyroid stimulating hormone (thyrotropin).

The SHAP summary diagram reflects the contribution of each example to the optimal model output. The model prediction for small nodules was more inclined to a satisfactory curative effect, while that for the large nodules was more inclined to an unsatisfactory curative effect, as shown in **Figure 4**; however, some small nodules showed an unsatisfactory curative effect. A high thyroid stimulating hormone level, proportion of solid components < 20%, male sex, peripheral blood flow distribution, not high-risk, and peripheral and internal blood flow distribution were more likely to predict a satisfactory curative effect. A proportion of solid components ≥80%, proportion of solid components 50%–80%, and a higher blood flow score tended towards unsatisfactory.

## 4 Discussion

In this study, six machine-learning methods were used to construct models to predict the curative effect of benign thyroid nodule ablation. The accuracy and AUC values ranged from 69.2% to 78.9% and 0.77 to 0.86, respectively. Among them, the XGBoost algorithm had the highest AUC value, confirming that machine learning predicted the curative effect.

In previous studies, traditional statistical methods, such as logistic regression, were usually employed to predict the curative effect of thyroid nodule ablation (12, 13). The advantage of traditional statistical methods is that they provide an interpretable and verifiable way to analyze data, but they require high data, meeting certain assumptions and conditions, and may not perform well on nonlinear and high-dimensional data. The advantage of machine learning is that it can handle large-scale and high-dimensional data, automatically adjust the model according to changes in data, and improve the accuracy of prediction and analysis (14).

Negro et al. (8) first used machine learning to build a model based on ultrasonic characteristics to identify benign thyroid nodules with 12-month efficacy ≥50% after ablation. The prediction accuracy of the model reached 85%. In this study, clinical features were also included, and various algorithms were used to build models. XGBoost had the best performance and the highest AUC. XGBoost is an algorithm based on the decision tree developed by Chen Tianqi et al. (15), which can upgrade multiple weak predictors into a stronger predictor. Because of its high accuracy, strong flexibility, and over-fitting, XGBoost was considered a competitive alternative to regression analysis and has been successfully used to predict various clinical results (16, 17). The disadvantage was that it was time-consuming and space-complex. In addition, LGBM and RF also showed superior accuracy and discrimination. Therefore, for those who need rapid modeling and prediction, and the dataset is not very large, RF or LGBM can be considered.

However, machine learning models often have the “black box” characteristic, and it was difficult to explain the internal mechanism of the model and the basis of prediction results. SHAP is a method based on game theory, which can quantify the contribution of each feature to the prediction results and provide an intuitive visual display (18). In this study, the XGBoost model was explained by SHAP. The important characteristics that affected VRR ≥70% were the proportion of solid components < 20%, initial nodule volume, blood flow score, peripheral blood flow, and the proportion of solid components 50%–80%.

The Shapley diagram demonstrated that the proportion of solid components < 20% had the greatest influence on the model output. Some studies have confirmed that the VRR of cystic nodules is significantly higher than that of solid nodules (19, 20). This is related to a reduction in nodule volume immediately after fluid aspiration. In addition, the initial nodule volume also had a significant influence on the output. Some studies have reported that the initial nodule volume is directly related to the VRR and that nodules with large initial volumes have a small VRR (21–23). There are two main reasons for the poor VRR of the greater tubercle: 1) The ablation reduction process involves the removal of degenerative and necrotic tissue (24); there are more degenerative and necrotic tissues in the greater tubercle, a long removal time, and slow tubercle shrinkage. 2) The larger the nodules, the closer the relationship with the surrounding vital organs and tissues, the wider the safe area preserved around the nodules during ablation, and the higher the probability of regeneration (25). Therefore, large nodules usually require supplementary ablation (26) or even secondary operation (27). However, some small nodules have an unsatisfactory curative effect, which may be related to the fact that the ablation range of small nodules was too large, exceeding the initial nodule volume, resulting in an insignificant reduction in postoperative VRR.

In this study, the higher the blood flow score, the more likely it was to predict an unsatisfactory curative effect; this was considered to be related to the “heat sink effect” caused by abundant blood flow around the lesion during ablation (28). In addition, a peripheral blood flow distribution pattern tended to predict a satisfactory curative effect, which may be because the blood flow around the nodule is beneficial to clear the necrotic tissue of the ablation focus.

Generally, during the ablation of high-risk nodules, the operator narrows the ablation range to avoid damaging the surrounding important structures, resulting in incomplete ablation and *in situ* recurrence (29). In this study, the nodules in high-risk locations had little negative influence on the VRR, likely related to the “lever separation technique” and “fluid isolation” methods adopted in the ablation strategies to improve efficacy and reduce the occurrence of complications (30).

This study had several limitations. First, 518 samples may not optimally train a machine learning model, and there was no external verification. Second, the main features that had a significant impact on the output came from ultrasonic images, which were extracted manually, and ultrasound omics was not applied to extract and analyze additional image features. In future studies, we will standardize image storage and use ultrasound omics to extract and analyze ultrasonic image features.

## 5 Conclusion

An interpretable machine learning model was developed to predict the efficacy of benign thyroid nodule ablation. This was a preliminary exploration of machine learning with a gap in actual clinical applications. Therefore, more in-depth research should be conducted to implement machine-learning models that can serve clinics more accurately.

## Data Availability

The raw data supporting the conclusions of this article will be made available by the authors, without undue reservation.

## Supplementary material

Supplementary Figure 1 Machine learning model construction and prediction. LR, logistic regression; SVM, support vector machine; DT, decision tree; RF, random forest; XGBoost, eXtreme Gradient Boosting; LGBM, Light Gradient Boosting Machine; AUC, area under curve of the receiver operating characteristic; SHAP, SHapley Additive exPlanations.
