# Supplementary figures and images for: A prognostic model for thermal ablation of benign thyroid nodules based on interpretable machine learning
Source: Front Endocrinol (Lausanne). 2024 Aug 19;15:1433192. doi: 10.3389/fendo.2024.1433192 (PMC11366643; doi:10.3389/fendo.2024.1433192)

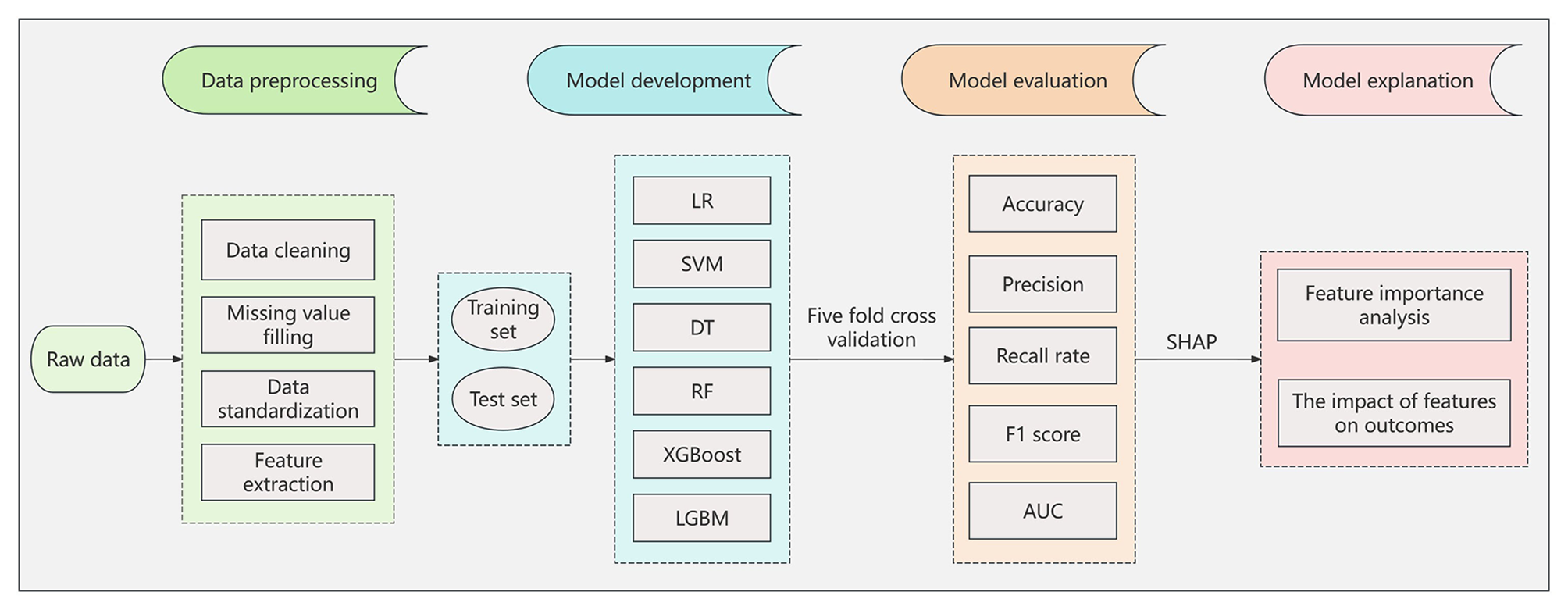

Supplement: Supplementary Figure 1 — Machine learning model construction and prediction. LR, logistic regression; SVM, support vector machine; DT, decision tree; RF, random forest; XGBoost, eXtreme Gradient Boosting; LGBM, Light Gradient Boosting Machine; AUC, area under curve of the receiver operating characteristic; SHAP, SHapley Additive exPlanations. [file Image1.tif]
